# Unilateral orbital mass as the initial presentation of pediatric antineutrophil cytoplasmic antibody associated vasculitis: eosinophilic granulomatosis with polyangiitis versus granulomatosis with polyangiitis

**DOI:** 10.1186/s12348-026-00583-0

**Published:** 2026-04-22

**Authors:** Paulien Bonny, Ruben Van Paemel, Eva Schiettecatte, Jo Van Dorpe, Thomas Renson, Elke O. Kreps, Virginie G. S. Ninclaus

**Affiliations:** 1 Department of Ophthalmology, Ghent University Hospital, Ghent, Belgium; 2 Department of Internal Medicine and Pediatrics, Ghent University Hospital, Ghent, Belgium; 3 Department of Radiology, Ghent University Hospital, Ghent, Belgium; 4 Department of Anatomical Pathology, Ghent University Hospital, Ghent, Belgium

**Keywords:** Pediatric ANCA-associated vasculitis, Orbital mass, Orbital inflammation, Granulomatosis with polyangiitis, Eosinophilic granulomatosis with polyangiitis, MPO-ANCA.

## Abstract

**Background:**

Eosinophilic granulomatosis with polyangiitis (EGPA), formerly known as Churg-Strauss syndrome, is a rare antineutrophil cytoplasmic antibody (ANCA)–associated vasculitis characterized by asthma, eosinophilia, and systemic vasculitis. Ocular involvement is uncommon (6–20%) and usually follows systemic disease. In contrast, granulomatosis with polyangiitis (GPA) more frequently (30–60%) involves the orbit and may present as isolated orbital disease. Distinguishing EGPA from GPA can therefore be challenging when orbital inflammation is the initial manifestation, particularly in children.

**Case presentation:**

A 7-year-old girl was referred with unilateral orbital swelling that was initially treated as preseptal cellulitis with oral amoxicillin and topical ciprofloxacin, without clinical improvement. Examination revealed a firm, mobile, tender superotemporal orbital mass with restricted upgaze, while visual acuity and fundus examination were normal. Orbital magnetic resonance imaging demonstrated an extraconal lesion exerting mass effect on the superior rectus muscle, globe, and lacrimal gland. The lesion was isointense on T1-weighted images, hyperintense on T2-weighted images, and showed marked contrast enhancement without diffusion restriction. Orbital biopsy revealed dense eosinophilic infiltration with granuloma formation and perivascular inflammation. Serologic testing demonstrated positive perinuclear (P-) ANCA with elevated myeloperoxidase (MPO) antibodies, supporting a diagnosis within the spectrum of ANCA-associated vasculitis. Systemic evaluation revealed no other inflammatory lesions, apart from bilateral otitis media with effusion. The patient underwent surgical debulking, which relieved the mass effect and provided diagnostic tissue. Subsequent treatment with high-dose systemic corticosteroids and rituximab resulted in clinical improvement with sustained systemic clinical and biochemical remission. Follow-up MRI, however, showed no significant reduction in the size of the orbital mass, necessitating a second, more extensive surgical debulking.

**Conclusion:**

Isolated orbital inflammation may represent an initial manifestation of ANCA-associated vasculitis in pediatric patients. Although eosinophil-rich granulomatous inflammation and MPO-ANCA positivity may suggest eosinophilic granulomatosis with polyangiitis, predominant orbital and ENT involvement in the absence of asthma or other atopic features may be more consistent with localized granulomatosis with polyangiitis. This case underscores the importance of early orbital biopsy, comprehensive serological evaluation, and cautious interpretation of residual orbital lesions within a multidisciplinary, longitudinal diagnostic approach.

## Background

Antineutrophil cytoplasmic antibody (ANCA)–associated vasculitides are rare autoimmune disorders characterized by necrotizing inflammation of small- to medium-sized blood vessels. This group includes granulomatosis with polyangiitis (GPA), microscopic polyangiitis, and eosinophilic granulomatosis with polyangiitis (EGPA). Although these conditions share overlapping clinical, serological, and histopathological features, they differ in organ involvement, disease course, and prognosis [1, 2]. 

EGPA is classically characterized by asthma, peripheral eosinophilia, and systemic vasculitis, often progressing through distinct clinical phases. Starting with an atopic phase (asthma and rhinitis), followed by an eosinophilic infiltration phase (eosinophils accumulate in tissues such as the lungs or skin, causing local damage) and eventually a vasculitic phase (with granuloma formation). Ocular involvement is uncommon and typically occurs after systemic disease onset [3]. In contrast, ocular and orbital manifestations are well recognized in GPA and may represent the initial or even isolated presentation, particularly in limited forms of the disease [1, 2]. In children, ANCA-associated vasculitides are exceptionally rare and isolated orbital involvement poses a significant diagnostic challenge [1, 4]. We report a pediatric case of unilateral orbital inflammation initially classified as EGPA, in which subsequent reassessment favored localized GPA, illustrating the diagnostic overlap between these entities.

## Case presentation

A 7-year-old girl was urgently referred to the ophthalmology department with progressive, fluctuating swelling and redness of the left upper eyelid, initially suspected to be preseptal cellulitis. There was no history of trauma. Previous treatment with amoxicillin orally and ciprofloxacin eye drops had no effect. Clinical examination revealed a palpable, mobile mass superior to the left levator palpebrae superioris muscle. Visual acuity was 20/20 bilaterally. The patient did not report diplopia. Ocular motility was largely preserved, although a mild apparent limitation in upgaze was noted clinically, most likely related to local swelling and mass effect of the lesion. Orthoptic examination demonstrated full ocular motility without a measurable ocular deviation. Systemic evaluation included a complete pediatric physical examination, laboratory analysis, chest CT imaging, spirometry, urinalysis, and otorhinolaryngological assessment. Cardiac screening with electrocardiography and echocardiography was also performed. These investigations revealed no evidence of systemic vasculitic involvement, apart from bilateral otitis media with effusion leading to conductive hearing loss.

**Fig. 1:**
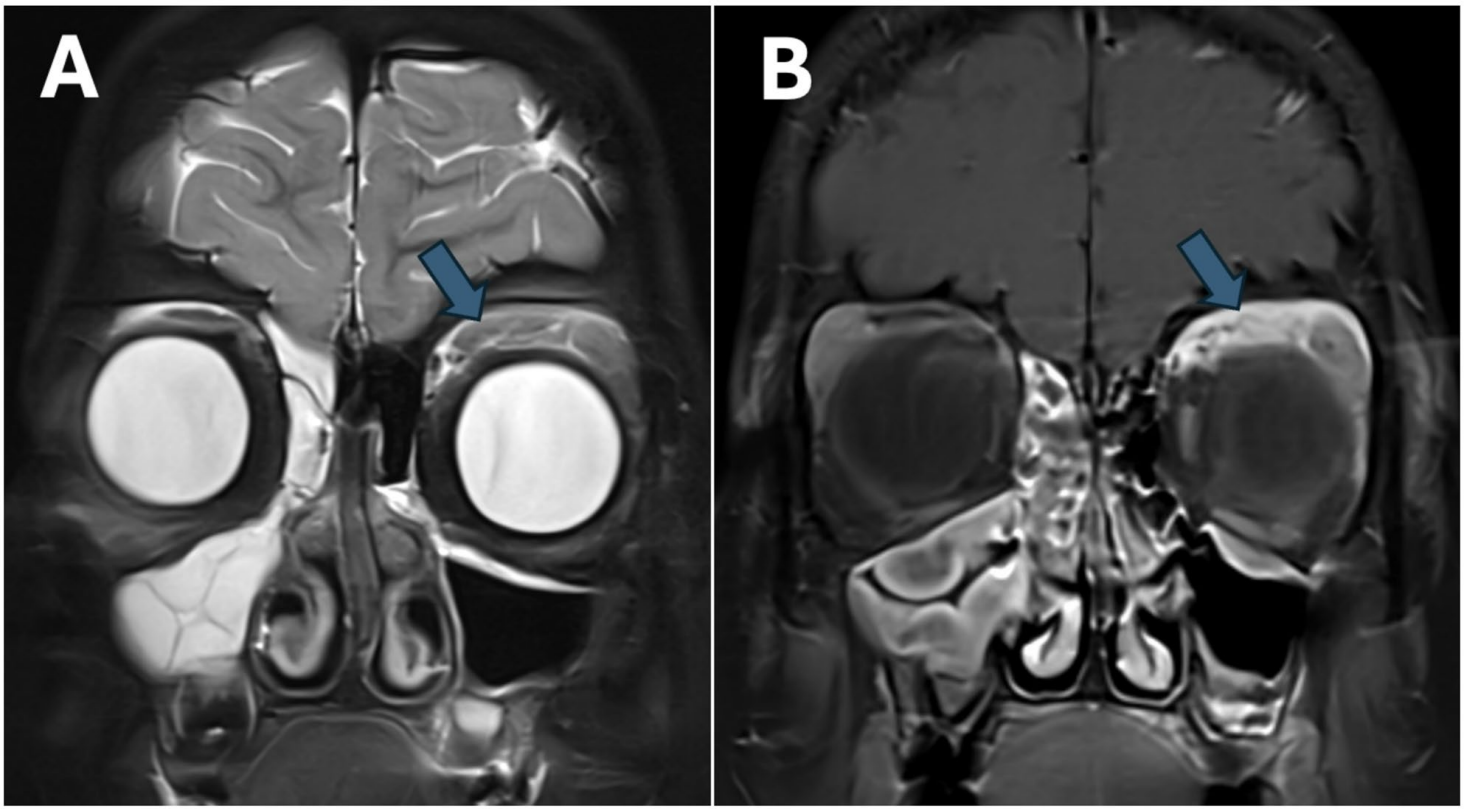
MRI: coronal T2-weighted turbo-inversion recovery magnitude (TIRM) image (**A**) and coronal T1-weighted image with fat saturation following gadolinium administration (**B**) demonstrate a T2-hyperintense mass with strong post-contrast enhancement (arrow)

MRI of the orbits demonstrated a well-circumscribed mass in the superior left orbit, predominantly intraorbital and extraconal, extending into the periorbital space. The lesion measured approximately 1.2 × 3.2 × 4.3 cm. Posteriorly it extended into the extraconal compartment, compressing the superior rectus muscle, anteriorly it exerted mass effect on the globe. Medially it contacted the superior oblique muscle, and laterally it displaced the lacrimal gland posteriorly toward the lateral rectus, with mild surrounding edema. No intracranial extension or bony invasion was present, and the contralateral orbit appeared normal. The lesion was mildly heterogeneous, demonstrated isointense signal on T1-weighted images and hyperintense signal on T2-weighted images, and showed intense contrast enhancement without diffusion restriction (Fig. 1). Dynamic contrast bolus imaging could not be evaluated because of premature vascular opacification. Moreover, MRI revealed an asymptomatic but important mucosal thickening of the paranasal sinuses indicative of sinusitis. Overall, findings were consistent with a left superior orbital mass with marked post-contrast enhancement. The main differential diagnoses included rhabdomyosarcoma and hemangioma.

**Fig. 2:**
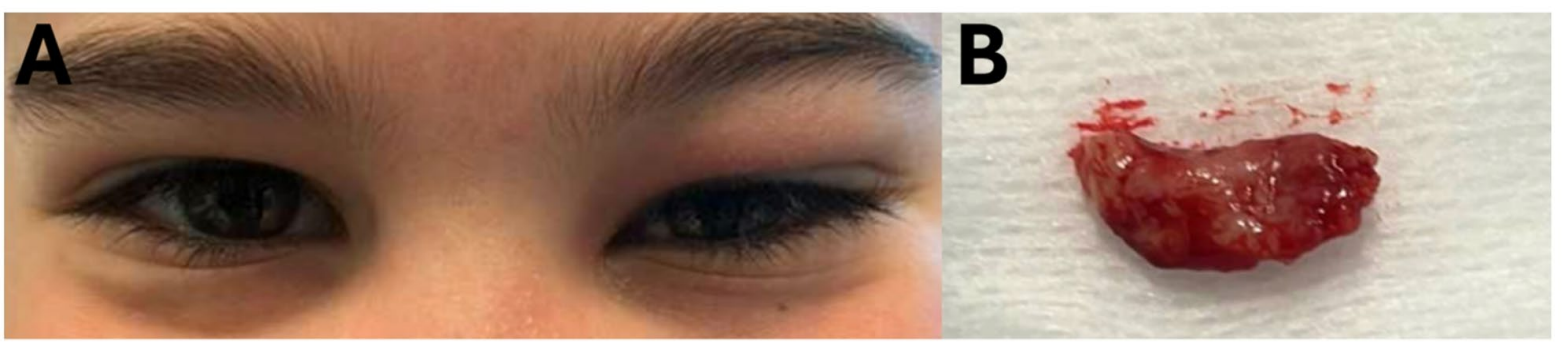
(**A**) F, 7Y: Clinical photograph at presentation showing left upper eyelid swelling. (**B**) Excised orbital tissue obtained after surgical debulking

**Fig. 3:**
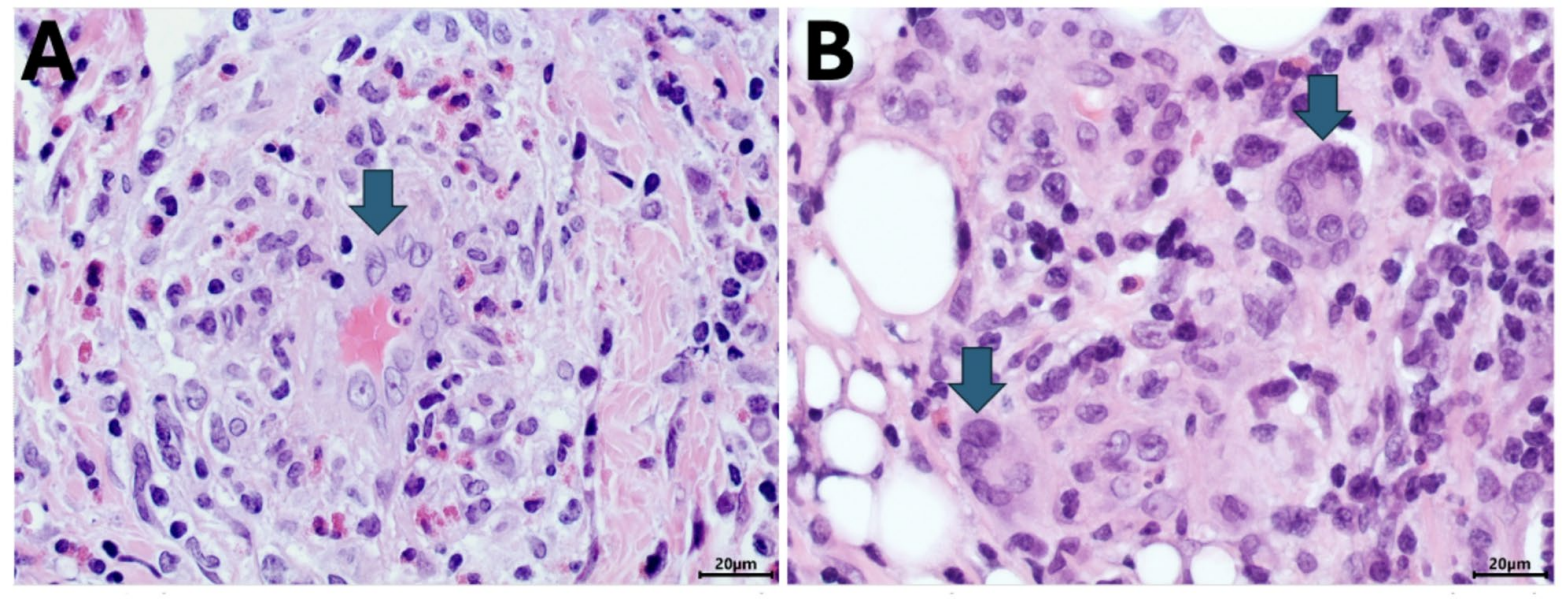
Pathology: (**A**) Small vessel showing an eosinophil-rich inflammatory infiltrate in its wall. The endothelium (arrow) is prominent and shows reactive changes, (**B**) Ill-defined granuloma showing two multinucleated giant cells (arrows)

An excisional biopsy was performed to obtain diagnostic tissue and to relieve mass effect (Fig. 2). Histopathological examination revealed highly vascularized fibro-adipose tissue with pronounced perivascular eosinophilic infiltration, an increased number of eosinophilic granulocytes and rare granulomas (Fig. 3). These findings were suggestive of either EGPA or GPA. Immunohistochemistry showed increased IgG4-positive plasma cells without diagnostic features of IgG4-related disease. Angiolymphoid hyperplasia with eosinophilia was considered, but molecular findings and the absence of lymphoma or sarcoma features argued against it.

Laboratory tests revealed a strongly positive perinuclear (P-) ANCA with elevated myeloperoxidase (MPO) antibodies (MPO-CLIA 122.7 U/mL; reference < 3.5 U/mL), while proteinase-3 antibodies (PR3) were negative (< 0.7 U/mL). Peripheral eosinophilia was 6% (normal < 5%). Antinuclear antibodies (ANA) were positive, suggestive of autoimmunity but not specific. Angiotensin-converting enzyme (ACE) was mildly elevated (79 U/L; normal < 65 U/L), a nonspecific finding sometimes seen in granulomatous disorders, although also in younger individuals. Immunophenotyping and bone marrow studies were unremarkable.

Based on the presence of eosinophil-rich granulomatous inflammation and MPO-ANCA positivity, a diagnosis within the spectrum of ANCA-associated vasculitis was established, most consistent with granulomatosis with polyangiitis, given the clinical presentation with an orbital mass, otitis media, and sinusitis, and the absence of asthma or other atopic manifestations.

The patient was initially treated with systemic corticosteroids (prednisolone 25 mg twice daily, corresponding to 2 mg/kg/day). After one month, the dose was reduced to 1 mg/kg/day, followed by a gradual taper over a total treatment duration of 4–5 months, guided by clinical response and treatment-related adverse effects. Rituximab was initiated as induction therapy (375 mg/m² once weekly for four consecutive weeks), with maintenance treatment planned at six-monthly intervals for at least two years.

Following surgical debulking and immunosuppressive therapy, the patient demonstrated marked clinical improvement with complete resolution of orbital pain and relief of the orbital mass effect. Follow-up MRI, however, showed a persistent superior intraorbital extraconal mass with extension toward the periorbital space, demonstrating intense contrast enhancement but only limited reduction in volume compared with the immediate postoperative imaging.

Given the persistence of a well-demarcated mass lesion radiologically distinguishable from the surrounding normal orbital tissue, and in order to differentiate between residual active inflammation and fibrotic scar tissue, a second, macroscopic complete surgical debulking was therefore performed, extending posteriorly beyond the equator toward the superior oblique muscle and the lacrimal gland, resulting in complete macroscopic removal of the lesion. Histopathological examination demonstrated predominantly fibrotic tissue without evidence of active vasculitis. At the time of submission, complete macroscopic resection had been achieved without histological evidence of active vasculitis, and the patient remains in clinical and biochemical remission. Maintenance therapy with rituximab is planned every six months for a total duration of two years. The patient is currently followed clinically every three months and will undergo long-term follow-up in the pediatric rheumatology clinic to monitor for potential relapse or evolution toward systemic disease. Sequential MRI surveillance is planned at six-month intervals as part of structured multidisciplinary follow-up.

## Discussion

Here we presented the case of a 7-year-old girl with an intraorbital mass. The differential diagnosis includes benign and malignant neoplasias, vascular and inflammatory etiologies [4]. Rhabdomyosarcoma, the most common primary malignant orbital tumor in children, must be ruled out first. Infantile hemangiomas were included in the differential diagnosis given their prevalence (4–5%) as benign vascular tumors in childhood. They typically appear within the first 1–2 months of life, grow rapidly during the first year and gradually regress over time. Lymphangiomas (lymphatic malformations) were also considered, although rare, representing approximately 0.3% to 4% of all orbital tumors. These congenital, non-involuting vascular malformations often present with intermittent or progressive unilateral orbital swelling, sometimes exacerbated by infection or hemorrhage [5]. Lymphoproliferative disorders such as orbital lymphoma were also contemplated, despite being rare in pediatrics. Additional considerations included idiopathic orbital inflammatory disease, vascular malformations, and rare immune-mediated disorders or vasculitides. Given the wide range of potential causes, integrating clinical, serological, histopathological and immunohistochemical findings was essential [2, 5]. In this case, the combination of marked contrast enhancement on imaging, eosinophil-rich granulomatous inflammation on histopathology, and MPO-ANCA positivity supported ANCA-associated vasculitis as the most likely diagnosis.

This case illustrates the diagnostic complexity of isolated orbital inflammation as the first manifestation of ANCA-associated vasculitis in a pediatric patient. Although initial clinicopathological findings supported a diagnosis of EGPA, several features argue against this classification. EGPA is typically associated with asthma, allergic disease and marked peripheral eosinophilia, often preceding vasculitic manifestations. Ocular involvement occurs in only 6–20% of cases and usually develops after systemic disease onset [3]. In contrast, orbital involvement is a well-recognized feature of GPA, occurring in up to 30–60% of patients, and may represent the initial or isolated manifestation, particularly in limited forms of the disease. While MPO-ANCA positivity and eosinophilic inflammation initially supported EGPA, MPO-positive GPA is increasingly recognized and may show significant phenotypic overlap with EGPA, including eosinophil-rich infiltrates. Mild eosinophilia alone is not specific for EGPA and may be observed in other inflammatory or granulomatous conditions. Histopathological findings of granulomatous inflammation with perivascular involvement are also not exclusive to EGPA and may be seen in GPA, particularly in localized disease. Rare cases of limited GPA presenting with isolated orbital involvement and eosinophilic features have been reported, underscoring the overlap between these entities [1, 2]. In the absence of asthma, allergic disease, or systemic involvement, and given the predilection of GPA for orbital disease, this presentation is more consistent with localized MPO-positive GPA with eosinophilic features rather than true EGPA.

A limited number of pediatric reports describe orbital swelling as the initial presentation of an ANCA-associated vasculitis. Baker et al. presented an 8-year-old girl with orbital pseudotumor as the first manifestation of EGPA [6]. Zhu et al. reported bilateral orbital inflammation in a child with EGPA [7]. Saad et al. described an 11-year-old girl with unilateral orbital swelling as the presenting symptom of GPA, initially suspected as IgG4-related disease [8]. 

These cases underline the clinical variability and diagnostic overlap between EGPA, GPA, and other immune-mediated disorders, particularly in the absence of systemic features. An overview of this overlap is highlighted in Table 1.

**Table 1:** This table highlights the overlapping yet distinct clinical, serological, and histopathological features of eosinophilic granulomatosis with polyangiitis (EGPA) and granulomatosis with polyangiitis (GPA), with EGPA typically associated with asthma and eosinophilia and GPA more often presenting with orbital involvement and limited disease [2, 3]

Feature	EGPA	GPA
Asthma / allergic disease	Present	Absent
Peripheral eosinophilia	Mild	Possible
MPO-ANCA positivity	Typical (35–40%)	Recognized (10–20%)
Orbital involvement	Rare	Common
Isolated disease	Unusual	Typical for limited GPA
Histology (eosinophils, granulomas)	Compatible	Compatible
Pediatric presentation	Very rare	Rare but reported

This case highlights that ANCA-associated vasculitides exist along a clinical and pathological spectrum, particularly in pediatric patients and that disease classification may evolve over time, emphasizing the importance of longitudinal follow-up [1]. 

Although EGPA and GPA belong to the spectrum of ANCA–associated vasculitides, their underlying pathophysiology and long-term management strategies differ. EGPA is predominantly driven by eosinophilic inflammation and Th2-mediated immune responses, although granulomatous inflammation and necrotizing vasculitis can also occur, whereas GPA is primarily characterized by granulomatous inflammation and necrotizing vasculitis. This distinction has implications for both disease phenotype and targeted therapy. Consequently, therapeutic approaches diverge beyond the acute phase, although initial management may overlap in cases of organ-threatening disease, including orbital involvement [9, 10]. 

Systemic corticosteroids form the cornerstone of initial treatment in both EGPA and GPA. In EGPA, additional therapy is often tailored toward eosinophil-driven disease, particularly in patients with asthma and marked peripheral eosinophilia. Steroid-sparing agents such as methotrexate or azathioprine may be used in non–organ-threatening disease, while biologic therapies targeting interleukin-5, such as mepolizumab, have demonstrated efficacy in reducing disease activity and relapse rates in EGPA [9, 11]. Rituximab is generally reserved for patients with vasculitic or organ-threatening manifestations.

In contrast, management of GPA, including limited forms with isolated orbital involvement, primarily targets granulomatous and vasculitic inflammation. In addition to systemic corticosteroids, immunosuppressive therapy with rituximab or cyclophosphamide is recommended for induction of remission, with rituximab increasingly favored in pediatric patients due to its efficacy and safety profile [10]. Surgical intervention, such as orbital biopsy or debulking, may be required for diagnostic confirmation or relief of mass effect in localized orbital disease. In pediatric patients presenting with isolated orbital disease, initial therapeutic strategies for EGPA and GPA therefore largely overlap, as reflected in the present case. However, accurate disease classification remains essential for guiding long-term management, anticipating disease evolution, tailoring follow-up, and assessing the risk of relapse or systemic involvement.

This report is limited by the relatively short duration of follow-up after the second surgical debulking. At the time of submission, postoperative MRI surveillance has not yet been performed and additional immunosuppressive therapy is planned. Consequently, the long-term radiological evolution and durability of disease control cannot yet be fully assessed.

An additional limitation relates to the early initiation of immunosuppressive therapy. In both eosinophilic granulomatosis with polyangiitis and granulomatosis with polyangiitis, ocular manifestations may precede systemic disease. Early treatment with corticosteroids and rituximab may theoretically suppress the subsequent development of systemic features that could further clarify the underlying disease phenotype. Continued long-term follow-up is therefore essential to monitor for potential evolution toward systemic disease.

## Conclusion

In conclusion, isolated orbital inflammation may represent an initial manifestation of ANCA-associated vasculitis in pediatric patients. In the present case, eosinophil-rich granulomatous inflammation and MPO-ANCA positivity initially raised concern for eosinophilic granulomatosis with polyangiitis. However, the clinical presentation with predominant orbital and ENT involvement and the absence of asthma or other atopic features were more consistent with localized granulomatosis with polyangiitis.

At the time of the second procedure, findings were consistent with complete local remission, as no active vasculitis was identified histologically and no clinical signs of orbital inflammation were present. Further immunosuppressive treatment and radiological follow-up are planned. This case highlights the importance of early orbital biopsy, comprehensive serological evaluation, and cautious interpretation of residual orbital lesions. In selected cases, surgical debulking beyond diagnostic biopsy may be valuable not only therapeutically but also diagnostically, as it can help differentiate persistent active inflammation from fibrotic scar tissue. Multidisciplinary, longitudinal follow-up remains essential to refine diagnosis and guide management within the spectrum of pediatric ANCA-associated vasculitis.

## Data Availability

All data generated or analyzed during this case report are included in this published article. No additional datasets were generated or analyzed. Further details are not publicly available due to patient confidentiality.

## Abbreviations

ACE: Angiotensin-converting enzyme
ANA: Antinuclear antibodies
ANCA: Antineutrophil cytoplasmic antibody
CLIA: Chemiluminescent immunoassay
EGPA: Eosinophilic granulomatosis with polyangiitis
GPA: Granulomatosis with polyangiitis
IgG4: Immunoglobulin G4
MPO: Myeloperoxidase
MRI: Magnetic resonance imaging
P-ANCA: Perinuclear antineutrophil cytoplasmic antibody
PR3: Proteinase-3
TIRM: Turbo inversion recovery magnitude
